# Brain RNA-Seq Profiling of the Mucopolysaccharidosis Type II Mouse Model

**DOI:** 10.3390/ijms18051072

**Published:** 2017-05-17

**Authors:** Marika Salvalaio, Francesca D’Avanzo, Laura Rigon, Alessandra Zanetti, Michela D’Angelo, Giorgio Valle, Maurizio Scarpa, Rosella Tomanin

**Affiliations:** 1 Women’s and Children’s Health Department, University of Padova, Via Giustiniani 3, 35128 Padova, Italy; marika.salvalaio@unipd.it (M.S.); laura.rigon@unipd.it (L.R.); alessandra.zanetti@unipd.it (A.Z.); maurizio.scarpa@unipd.it (M.S.); rosella.tomanin@unipd.it (R.T.); 2 Pediatric Research Institute—Città della Speranza, Corso Stati Uniti 4, 35127 Padova, Italy; 3 CRIBI Biotechnology Center, University of Padova, Viale G. Colombo 3, 35121 Padova, Italy; michela.dangelo@unipd.it (M.D.); giorgio.valle@unipd.it (G.V.); 4 Brains for Brain Foundation, Via Giustiniani 3, 35128 Padova, Italy

**Keywords:** RNA-seq, Hunter syndrome, lysosomal storage disorders, neurodegenerative diseases, axon guidance, calcium homeostasis, synapse, circadian rhythm, Wnt signaling, neuroinflammation

## Abstract

Lysosomal storage disorders (LSDs) are a group of about 50 genetic metabolic disorders, mainly affecting children, sharing the inability to degrade specific endolysosomal substrates. This results in failure of cellular functions in many organs, including brain that in most patients may go through progressive neurodegeneration. In this study, we analyzed the brain of the mouse model for Hunter syndrome, a LSD mostly presenting with neurological involvement. Whole transcriptome analysis of the cerebral cortex and midbrain/diencephalon/hippocampus areas was performed through RNA-seq. Genes known to be involved in several neurological functions showed a significant differential expression in the animal model for the disease compared to wild type. Among the pathways altered in both areas, axon guidance, calcium homeostasis, synapse and neuroactive ligand–receptor interaction, circadian rhythm, neuroinflammation and Wnt signaling were the most significant. Application of RNA sequencing to dissect pathogenic alterations of complex syndromes allows to photograph perturbations, both determining and determined by these disorders, which could simultaneously occur in several metabolic and biochemical pathways. Results also emphasize the common, altered pathways between neurodegenerative disorders affecting elderly and those associated with pediatric diseases of genetic origin, perhaps pointing out a general common course for neurodegeneration, independent from the primary triggering cause.

## 1. Introduction

Lysosomal storage disorders (LSDs) are a group of inherited metabolic syndromes, sharing the inability to degrade specific endolysosomal substrates. LSDs are monogenic and due to genetic alterations in housekeeping genes, therefore the enzyme deficit results in failure of several biochemical activities in many tissues and organs, including brain in the severe forms of the disorders.

Hunter syndrome (MPS II, OMIM 309900) is one of the most common mucopolysaccharidoses, a subgroup of LSDs. It is an X-linked recessive disease caused by the deficit of the lysosomal hydrolase iduronate 2-sulfatase (IDS, EC3.1.6.13) involved in the catabolism of two mucopolysaccharides (or glycosaminoglycans, GAGs), heparan- and dermatan-sulfate. The accumulation of undegraded GAGs in lysosomes affects functions of most cell types, tissues and organs, including viscera, skeleton, connective tissue and central nervous system (CNS). The severe forms, accounting for about seventy-five percent of Hunter cases, present with major impairment of cognitive skills and delay/regression of mental development [1]. In these patients, in the early years of life, behavioral problems are common, including defiance, aggression and hyperactivity [1].

The pathophysiology of Hunter syndrome, as the complex mechanism somehow linking the accumulation of GAGs to the subsequent course of the disease, is not yet clear. During the last years, the traditional view of lysosome as a simple cell waste processor has been replaced by a more dynamic view of this organelle, which seems to be a key node of different interconnected cellular pathways regulating cell metabolism and homeostasis [2]. Whether this scenario also applies to CNS involvement remains obscure. However, the current priority of clinicians and researchers involved in MPSs, and in LSDs in general, is to treat the CNS disease, given the inefficacy of the presently available recombinant enzymes on this district, due to their inability to cross the blood-brain barrier. The understanding of the brain disease pathophysiology, the identification of new, potential therapeutic targets as well as disease biomarkers, would be of great help to reach this goal.

The purpose of this work was to perform, for the first time in Hunter syndrome, a wide characterization of two macro-area of the MPS II mouse model using RNA sequencing technology (RNA-seq). The first macro-area is the cerebral cortex (Cx), while the second one includes the midbrain, the diencephalon and the hippocampus (M).

The Ids-ko mouse used in these experiments has been extensively studied and characterized by different research groups, including ours. As for the CNS involvement, biochemical and histological analyses demonstrated both primary storage (i.e., glycosaminoglycans) [3] and secondary storage (GM2 and GM3 gangliosides [4], and α-synuclein [5]) together with vacuolization, apoptosis, degeneration of neurons and glial cells, inflammation and astrogliosis in different brain areas [3]. Moreover, behavioral tests confirmed a cognitive decline with alteration of spatial working memory, response to novel environments, anxiety-related behavior, coordination and balance, mainly starting from eight months of age [3].

Previously, characterization of brain areas using a transcriptomic approach had been performed in the cerebral cortex of MPS I and MPS IIIB animal models, the first ever analyzed almost 15 years ago [6]. Afterwards, a shotgun proteomics analysis was carried out in the hippocampus of the MPS I mouse model [7] while, for MPS VII, the analysis was first conducted by using a microarray approach [8] and recently extended, by applying both microarray and proteome analyses, to the hippocampus area [9]. In relation to the gene expression brain profile described for these disorders, our study confirms the involvement of calcium signaling, neuroinflammation, neurodegeneration, circadian rhythm and axon guidance, with neuroinflammation showing different alterations between the two areas.

As for Mucopolysaccharidosis type II, this study represents an important starting-point for the comprehension of its CNS pathophysiology highlighting, among others, impairment of the synaptic transmission pathways and of the Wnt signaling, crucial molecules and mechanisms to target and/or modulate new potential therapeutic strategies.

## 2. Results

### 2.1. Reads and Quality Control

RNA samples isolated from cerebral cortex (Cx) and midbrain/diencephalon/hippocampus (M) of seven IDS knock-out (Ids-ko) and seven wild type (wt) mice were analyzed as described in Materials and Methods Section. They are codified as follows: CxH = cerebral cortex from Ids-ko mice; MH = midbrain/diencephalon/hippocampus from Ids-ko mice; CxWT = cerebral cortex from wt mice; MWT = midbrain/diencephalon/hippocampus from wt mice. Reads obtained from the RNA sequencing were subjected to alignment on the mouse genome, obtaining, on average, 56.5% of reads passing the quality control, 65.6% of which mapping to the annotated mouse genome (Table 1).

### 2.2. Differentially Expressed Genes

**Figure 1:**
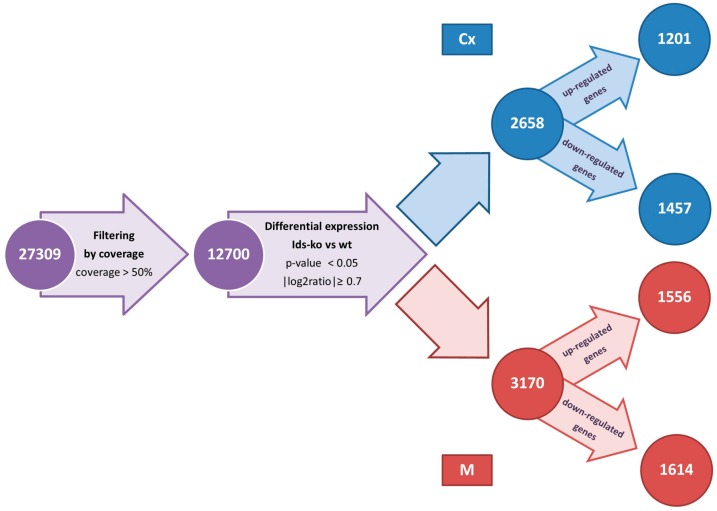
Flowchart showing the filtering process allowing identification of differentially expressed genes (DEG) in the comparison of Ids knock-out vs wild type mice for each brain area analyzed. Number of genes obtained in each step are reported in the circles. Cx = cerebral cortex; M = midbrain/diencephalon/hippocampus.

The 27,309 mouse genes thus annotated underwent appropriate filtering and the differentially expressed genes (DEG) have been identified for the following comparisons: CxH vs. CxWT, MH vs. MWT, CxH vs. MH and CxWT vs. MWT. The top 30 up-regulated and down-regulated genes for each comparison, are reported in Table S1. From the comparison of Ids-ko vs. wt samples, 1201 and 1556 up-regulated genes and 1457 and 1614 down-regulated genes in Cx and M, respectively, were identified (Figure 1).

As shown in Table S1, in Ids-ko compared to wt mice the degree of down-regulation is much higher than that of up-regulation, indicated by the reported fold change.

**Figure 2:**
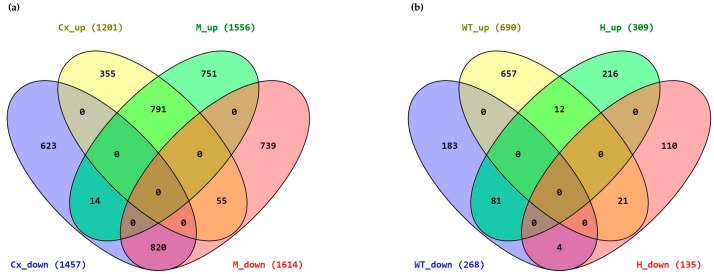
Venn diagrams of differentially expressed genes (DEG). (**a**) DEG for the comparisons of Ids knock-out (H) vs wild type (WT) mice in each area: CxH vs CxWT and MH vs MWT. The number of genes upregulated (Cx_up) and downregulated (Cx_down) for the comparison CxH vs CxWT, and upregulated (M_up) and downregulated (M_down) for the comparison MH vs MWT are reported in brackets ; (**b**) DEG for the comparison of cerebral cortex (Cx) vs midbrain/diencephalon/hippocampus (M) areas in each animal: CxWT vs MWT and CxH vs MH. The number of genes upregulated (WT_up) and downregulated (WT_down) for the comparison CxWT vs MWT, and upregulated (H_up) and downregulated (H_down) for the comparison CxH vs MH are reported in brackets. CxH = cerebral cortex from Ids knock-out mice; MH = midbrain/diencephalon/hippocampus from Ids knock-out mice; CxWT = cerebral cortex from wild type mice; MWT = midbrain/diencephalon/hippocampus from wild type mice.

As represented in the Venn diagrams (Figure 2a), in Ids-ko vs. wt mice, 791 up-regulated and 820 down-regulated genes are common between Cx and M, while 978 (355 + 623) and 1490 (751 + 739) genes are exclusive of each area. Notably, 14 and 55 genes, although common to Cx and M areas, turn out to have an opposite expression in the two areas (Table S2fa–). The evaluation of Cx vs. M (Figure 2b) came out with only 12 up-regulated and 4 down-regulated genes, in common between wt and Ids-ko mice, while 840 (657 + 183) and 326 (216 + 119) genes are peculiar of each area, this highlighting the loss of genes with an area specificity in the Ids-ko mouse; 81 and 21 genes are common between wt and Ids-ko mice, although showing an opposite expression (Table S3fa–).

In general, genes up-regulated in the Cx and down-regulated in the M area of the Ids-ko mouse are those predominantly involved in the inflammation and immune responses.

### 2.3. Gene Ontology (GO) Analysis

The differentially expressed genes obtained from the comparisons CxH vs. CxWT and MH vs. MWT were subjected to Gene Ontology (GO) analysis, by using DAVID Bioinformatics Resources 6.8 (Laboratory of Human Retrovirology and Immunoinformatics (LHRI), Frederick, MD, USA) [10].

**Figure 3:**
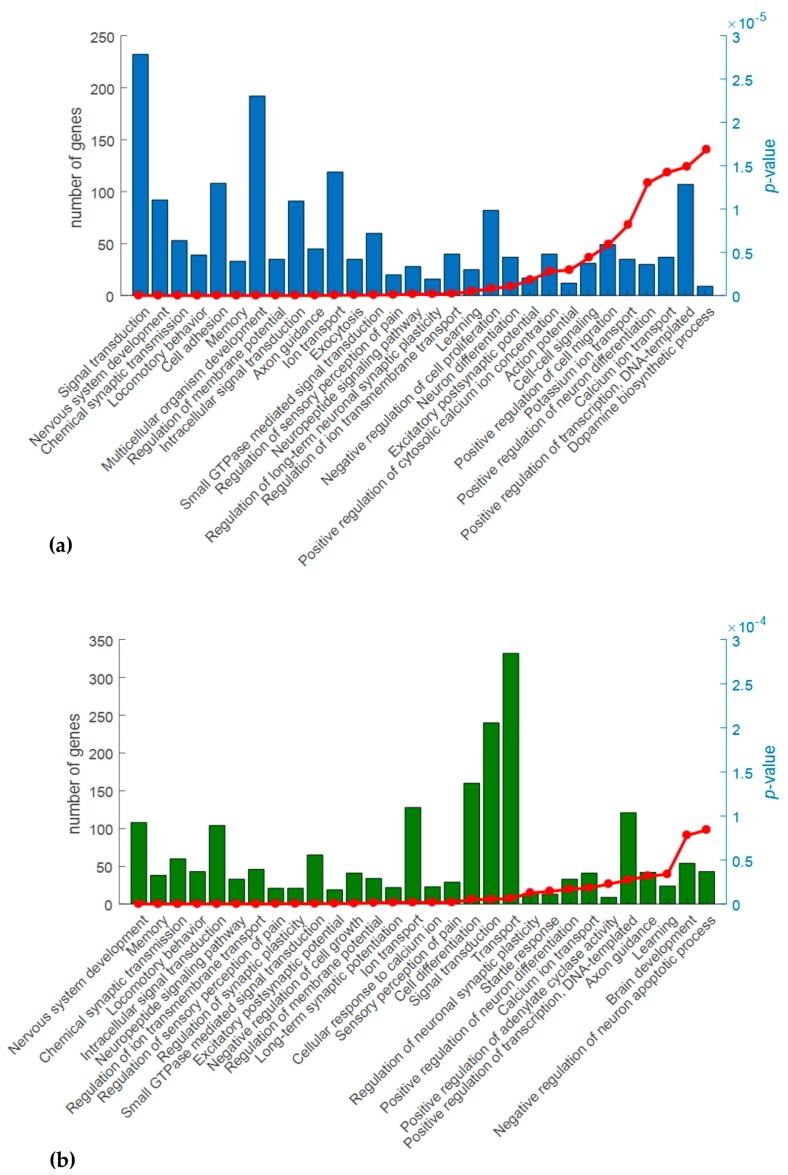
Analysis of Gene Ontology (GO) (Biological Process domain) of the differentially expressed genes (DEG) for the comparisons Ids knock out vs wild type mice in cerebral cortex (**a**) and midbrain/diencephalon/hippocampus (**b**). The graphs show for the top 30 over-represented GO terms (*X*-axis), the number of DEG included in each term (histogram bars, left *Y*-axis) and the *p*-values (red line, right *Y*-axis). *p*-Value ≤ 0.001.

DAVID software identified, in the Biological Process domain, 74 and 61 statistically significant terms (*p*-value ≤ 0.001), in Cx and M areas, respectively (Table S4a,b). Figure 3 shows the top 30 over-represented terms, among the most enriched of which are those involved in signal transduction, nervous system development, memory and chemical synaptic transmission. The analysis also highlighted other interesting categories, in terms of neurological impairment, such as axon guidance, synaptic plasticity, ion transport and neuropeptide signaling. All these categories are linked to calcium homeostasis and are implicated in the development of CNS.

**Figure 4:**
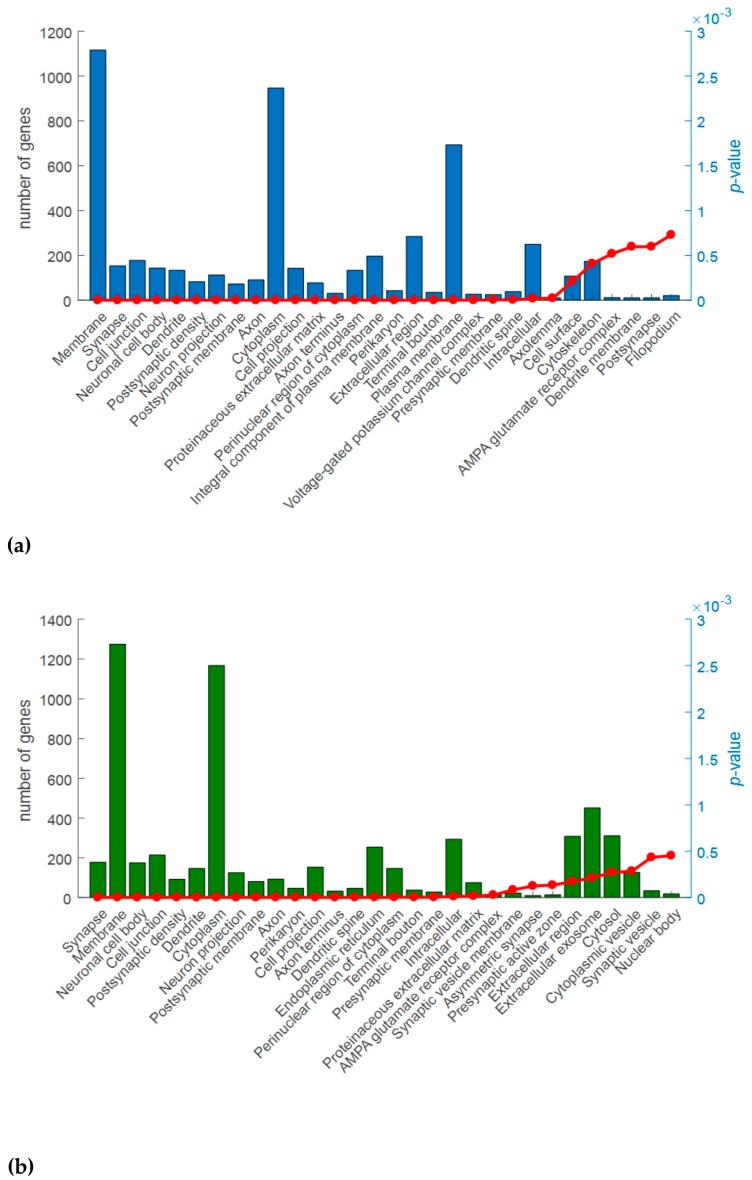
Analysis of Gene Ontology (GO) (Cellular Component domain) of the differentially expressed genes (DEG) for the comparisons Ids knock out vs wild type mice in cerebral cortex (**a**) and midbrain/diencephalon/hippocampus (**b**). The graphs show for the top 30 over-represented GO terms (*X*-axis), the number of DEG included in each term (histogram bars, left *Y*-axis) and the *p*-values (red line, right *Y*-axis). *p*-Value ≤ 0.001.

The Cellular Component domain identified 31 and 32 statistically significant terms in Cx and M areas, respectively (Figure 4, Table S4c,d). Among them, the most interesting terms are related to synapse, membrane, neuron projection, axon, cell junction, postsynaptic density, endoplasmic reticulum, voltage-gated channel and dendrite.

All these terms are involved in neuron plasticity with the class neuron projection, containing genes involved in the organization of neuronal projections by properly adjusting the cytoskeleton, and the calcium-dependent exocytotic vesicles.

**Figure 5:**
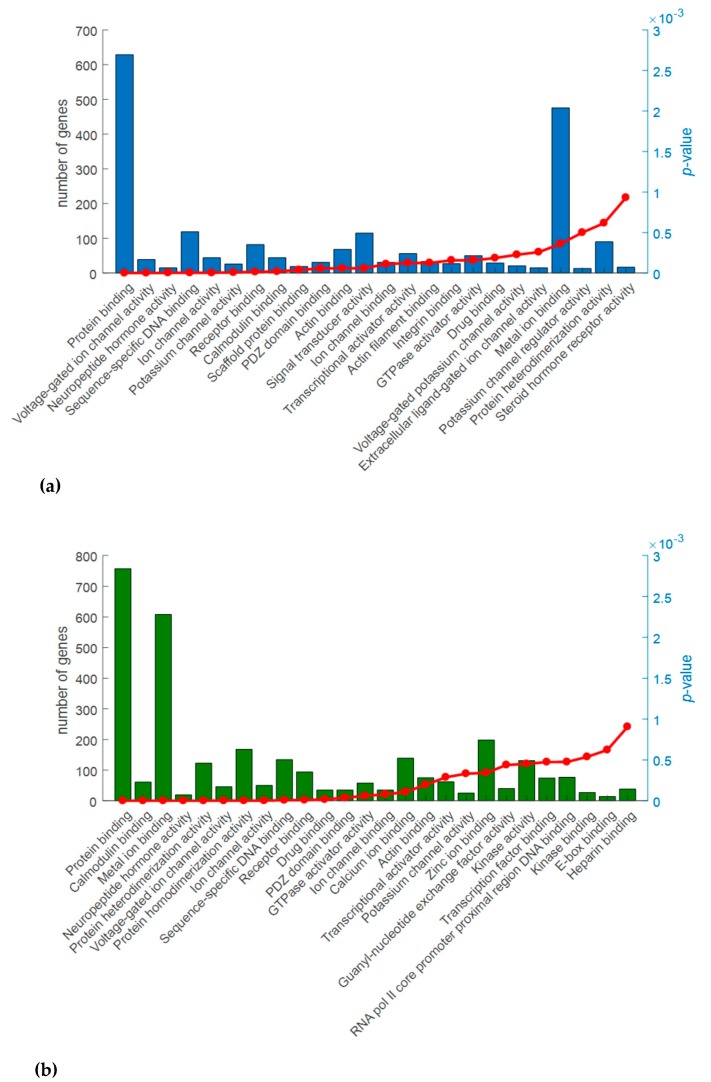
Analysis of Gene Ontology (GO) (Molecular Function domain) of the differentially expressed genes (DEG) for the comparisons Ids knock out vs wild type mice in cerebral cortex (**a**) and midbrain/diencephalon/hippocampus (**b**). The graphs show for each GO terms (*X*-axis), the number of DEG included in each term (histogram bars, left *Y*-axis) and the *p*-values (red line, right *Y*-axis). *p*-Value ≤ 0.001.

The Molecular Function domain identified 24 statistically significant terms in Cx and 26 in M (Figure 5), representative of each macro-area (Table S4e,f). Among them, the most interesting terms are related to protein binding, calmodulin binding, voltage-gated ion channel activity, receptor binding, GTPase activator activity and protein heterodimerization activity.

### 2.4. Pathway Analysis

**Figure 6:**
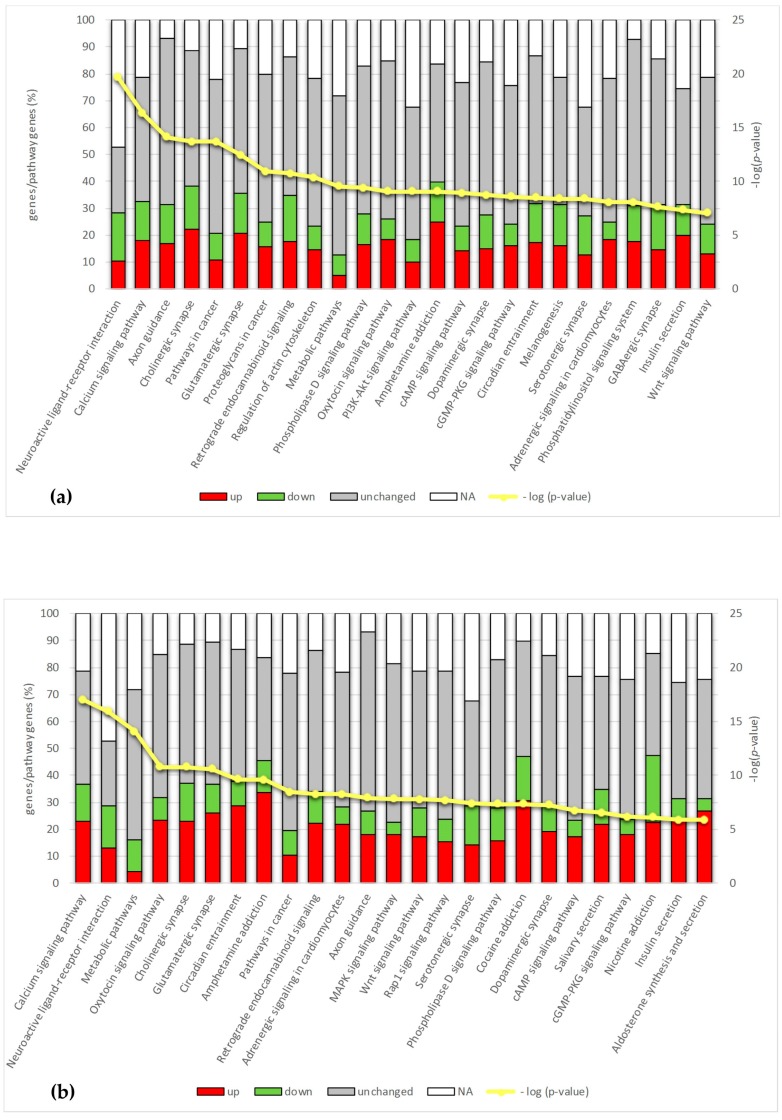
Pathways analysis by KEGG Orthology Based Annotation System. The top 25 most represented pathways in cerebral cortex (Cx) (**a**) and midbrain/diencephalon/hippocampus (M) (**b**). The bars include the following classes: up-regulated (red), down-regulated (green), unchanged (grey) and not available (white) genes. Data are expressed as percentage of genes present in the RNA-seq analysis vs. the total genes present in the pathway list: in Cx (**a**); and in M (**b**). The yellow line represents *p*-values (right *Y*-axis) (−log(*p*-value) ≥ 3 that corresponds to the *p*-value ≤ 0.001).

KEGG Orthology Based Annotation System (KOBAS) (Center for Bioinformatics, Peking University, Beijing, China) [11] was used to perform statistically enriched pathways analysis in the comparisons CxH vs. CxWT and MH vs. MWT. KOBAS identified 76 and 66 pathways statistically significant (*p*-value ≤ 0.001) in the Cx and M area, respectively (Table S5a,b). Figure 6a,b shows the top 25 most represented pathways in Cx and M, respectively.

Most of the pathways identified are involved in neuropeptide interactions and synaptic transmission. Calcium signaling was the second and the first represented pathway in Cx and in M areas, respectively. Moreover, this signaling interconnects several pathways as it includes genes involved in the neurotransmission (synapses), metabolic pathway, axon guidance and Wnt signaling, which result among the most enriched.

#### 2.4.1. Calcium Signaling

**Figure 7:**
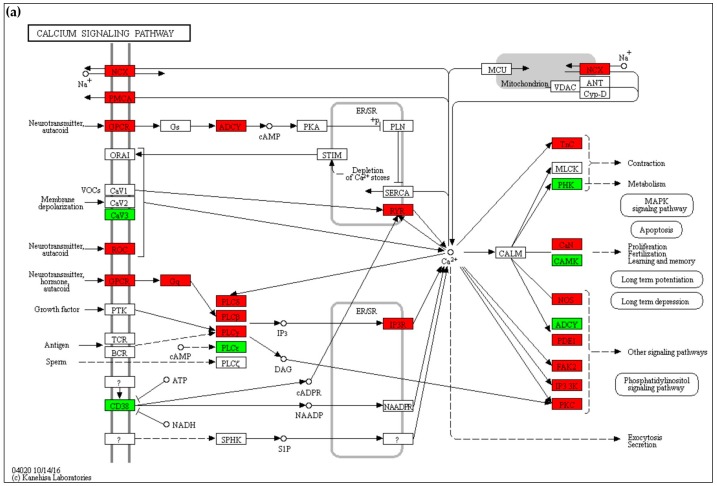

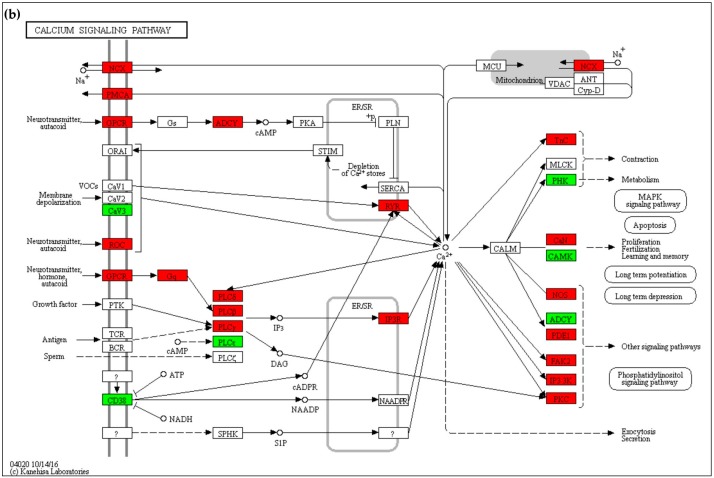
Calcium signaling maps (KEGG—mmu04020): up-regulated (red) and down-regulated (green) genes in the comparison Ids knock-out vs wild type mice in cerebral cortex (Cx) (**a**) and midbrain/diencephalon/hippocampus (M) (**b**) are shown. Genes are considered differentially expressed for |log2ratio| ≥ 0.7 and *p*-value < 0.05. ER/SR endoplasmic/sarcoplasmic reticulum; black arrow = molecular interaction or activation; dashed black arrow = indirect effect; ? = Unknown gene. For a description of the graphical notation, see Materials and Methods section.

The calcium signaling pathway is the most represented in the M area (67 DEG) and the second one in the cerebral cortex (59 DEG) of the MPS II mouse model (Table S6a). Severely compromised in both areas, among others it includes alterations of many genes related to calcium transporters of plasma membrane, mitochondrial membrane and endoplasmic reticulum, and to PI3K-Akt and cAMP signaling. Mainly in the M, but also in the Cx area, there is an alteration of the NMDA receptors for glutamate (*Grin*), the voltage-gated channels *Cacna*, most of the genes coding for the adenylate cyclase (*Adcy*), calcium/calmodulin-dependent protein kinase II (*Camk2*) and inositol 1,4,5-trisphosphate 3-kinase families (*Itpk*), and an up-regulation of *Slc8a2*, *Itpr1* and *Ryr* genes (Figure 7a,b).

#### 2.4.2. Synapse and Neuroactive Ligand–Receptor Interaction

**Figure 8:**
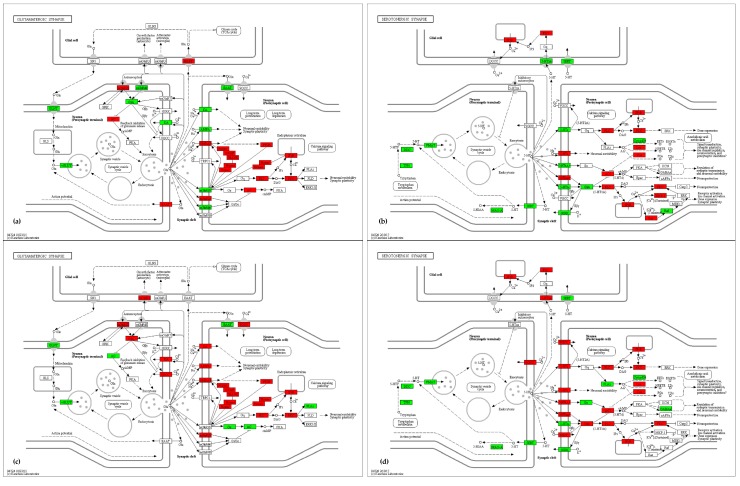
Glutamatergic and serotonergic synapses signaling maps (KEGG—mmu04724, mmu04726):up-regulated (red) and down-regulated (green) genes in the comparison Ids knock-out vs wild type mice in cerebral cortex (Cx) (**a**) and midbrain/diencephalon/hippocampus (M) (**b**) are shown. Genes are considered differentially expressed for |log2ratio| ≥ 0.7 and *p*-value < 0.05. Black arrow = molecular interaction or activation; dashed black arrow = indirect effect; white arrow = link to another map. For a description of the graphical notation, see Materials and Methods section.

This section describes the cholinergic, glutamatergic, dopaminergic, serotonergic and GABAergic synapse and neuroactive ligand–receptor interaction pathways (Table S6b). Most genes are differentially expressed, including *Agtr2*, *Calcr*, *Chrna3*, *Chrna6*, *Gabre*, *Gabrq*, *Glra1*, *Slc17a6*, *Slc18a2*, *Slc6a3*, *Slc6a4* and *Tph2.* Both areas show a significant alteration in the Ids-ko mouse model, and particularly the cerebral cortex. All gene families coding for the different kinds of receptors involved in chemical synapses result dysregulated: glutamatergic (*Grin* and *Grm*) (Figure 8a,c), GABAergic (*Gabr*), cholinergic (*Chrm* and *Chrn*) and adrenergic (*Adra*). Moreover, receptors for other types of low molecular weight neurotransmitters (*Glra*, *Htr*, and *Gng*) or peptide neurotransmitters (*Sstr*, *Tacr*, *Th*, *Thr*, *Tnn*, and *Vipr*) are altered. Channels for solutes (*Slc*), sodium (*Scn*) and potassium (*Kcn*) are also differentially expressed (Figure 8b–d).

#### 2.4.3. Axon Guidance

**Figure 9:**
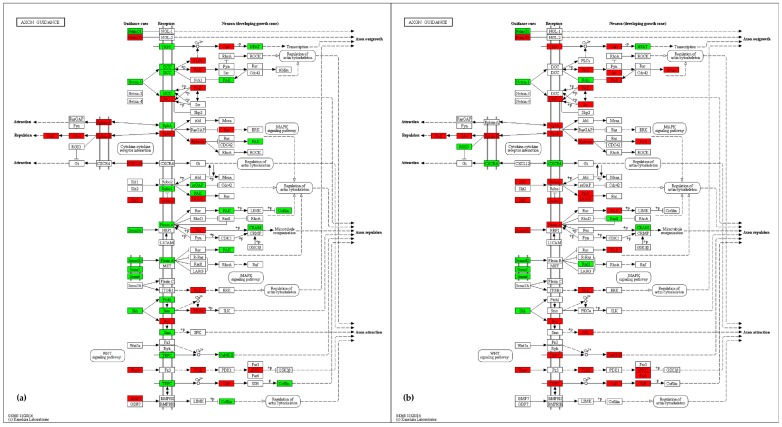
Axon guidance pathway maps (KEGG—mmu04360): up-regulated (red) and down-regulated (green) genes in the comparison Ids knock-out vs wild type mice in cerebral cortex (Cx) (**a**) and midbrain/diencephalon/hippocampus (M) (**b**) are shown. Genes are considered differentially expressed for |log2ratio| ≥ 0.7 and *p*-value < 0.05. Black arrow = molecular interaction or activation; dashed black arrow = indirect effect; white arrow = link to another map. For a description of the graphical notation, see Materials and Methods section.

Analysis of the axon guidance pathway reveals substantial changes in gene expression (Table S6c). The four families of signal molecules primarily involved in this pathway (netrins, ephrins, semaphorins, and slits) are all affected by significant variations. In particular, our analysis showed a down-regulation of netrins (*Ntn1*, *Ntng1*, and *Unc5c*) and semaphorins (*Sema4g*, *Sema5a*, *Sema6a*, and *Sema6d*) and an up-regulation of ephrins (*Efnb1*, *Efnb2*, *Ephb3*, and *Ephb6*) and slits (*Slit3*). In addition to these molecules, other factors involved in axon guidance, such as morphogens and genes involved in neurogenesis, are up- (*Wnt4*, *Bmp7*, *Pik3r2*, and *Robo3*) or down-regulated (*Shh*). Furthermore, as well as in other pathways, we here found up-regulated genes involved in the regulation of calcium, such as *Camk2a*, *Camk2b*, *Ppp3ca*, and *Trpc6* (Figure 9a,b).

#### 2.4.4. Circadian Rhythm and Entrainment

The circadian rhythm and entrainment pathway showed alterations only in the M area of the Ids-ko vs. the wt mice (Table S6d). We found an up-regulation of *Nr1d1*, *Bhlhe40*, *Cry2* and the periodic circadian clock genes (*Per1*, *Per2*, *Per3*) coding for crucial components of the circadian rhythms of locomotor activity, metabolism and behavior. Moreover, most of the genes involved in the circadian Ca^2+^ rhythms are altered both in Cx and M of the Ids-ko mice, such as adenylate cyclase genes (*Adcy1*, *Adcy5*, *Adcy7*, and *Adcy8*), calcium/calmodulin-dependent protein kinase II (*Camk2a* and *Camk2b*) and *Ryr*s.

#### 2.4.5. Regulation of Actin Cytoskeleton

Regulation of actin cytoskeleton (*Actn1*, *Actn4*) results partially impaired in both brain areas of the Ids-ko mouse model and mainly in the cortex (Table S6e). Signaling to cytoskeleton through G protein-coupled receptors (*Git1*) and integrins (*Itga3*, *Itga4*, *Itga5*, *Itga8* and *Itgb2*, *Itgb3*, *Itgb4*, *Itgb5*) is altered, as well as the activators of the Rho family of small GTPases, such as *Arhgef6* and p21 activated kinase family (*Pak3*, *Pak4*, *Pak6* and *Pak7*), which result differentially expressed. This reflects on proteins that directly regulate the organization of actin cytoskeleton, including *Cfl2* and the Arp2/3 complex (*Arpc1b*, *Arpc3*, and *Arpc5l*).

#### 2.4.6. Wnt Signaling

The Wnt signaling results perturbed in different steps of the pathway, both in Cx and in M (Table S6f). Wnt genes appear to be heavily up-regulated (*Wnt2*, *Wnt4*, *Wnt7b*, *Wnt9a*, and *Wnt10a*) and down-regulated (*Wnt2b*, *Wnt3*, and *Wnt9b*) in both areas as well as their receptors (*fzd1*/8) that result down-regulated, while the co-receptor *Lrp5* appears up-regulated only in Cx. The downstream genes in the canonical pathway (Wnt/β-catenin) involved in the so-called destruction complex (*Dvl*, *Ctnnb1*, *Apc*, *Gsk3b*, *Tcf*, *Lef*, and *Axin1/2*) do not appear altered; however, the transcriptional factors (*Tcf* and *Lef*) and other genes concerning the cell cycle (*Ppard*, *Ccnd1*, *Myc*, and *Jun*) are up-regulated only in the M area.

As for the non-canonical signaling, the Wnt/Pcp pathway mediated by *Map kinase*, *Rac* and *Rhoa*, is unchanged, whereas Wnt/Ca^2+^ pathway presents an altered expression of all the involved genes, in particular of phospholipase C (*Plcb1*), protein kinase C (*Prkca/b/g*), calcium/calmodulin-dependent protein kinase II (*Camk2a/2b*), which are up-regulated in both areas, while *Plcb4*, *Camk2d*, and the nuclear factor of activated T cells (*Nfatc2*) result down-regulated.

#### 2.4.7. Autophagy and Coordinated Lysosomal Expression and Regulation (CLEAR) Network

As shown by the pathway analysis performed with KOBAS, even a deeper manual analysis did not reveal major variations of the genes involved in the autophagy pathway (Table S6g). This pathway shows a poor dysregulation in both areas and only few genes are altered, including *Hmgb1*, *Stx17*, *Atg2a*, *Atg3*, *Atg10* and *Atg101*. Other genes, known as activators of autophagy, showed to be down-regulated (*Hif1a*, *Bnip3*, *Eif2a*, and *Rragb*) or up-regulated (*Camkk2* and *Tsc2*).

We also examined the expression changes of *Tfeb* and of the 73 genes belonging to the CLEAR (Coordinated Lysosomal Expression and Regulation) network, whose transcription is regulated by *Tfeb* itself [12]. The analysis evidenced no significant gene expression alterations for most genes, *Tfeb* included; only some hydrolases and accessory protein genes (*Gaa*, *Asah1*, *Ppt1*, *Gusb*, *Hexa*, *Ctsd*, and *Ifi30*), and few autophagy genes (*Hif1a* and *Rragb*/c) were dysregulated.

#### 2.4.8. Immune and Inflammatory Systems

As for astrogliosis, among the astrocyte-specific genes identified in vitro [13], some markers associated with signal transduction (*Mertk*) and cell metabolism (*Dio2* and *Ppp1r3g*) are up-regulated both in Cx and in M areas. However, *Aldh1l1*, considered the most specific marker gene for astrocytes in vitro [13], as well as other genes associated with astrocyte proliferation, hypertrophy and migration, such as *Olig2*, *Edn1* and *Stat3* [14,15] show to be unaltered in both areas. On the opposite, the early growth response 1 transcriptional factor (*Egr1*), involved in the scar formation and the related inhibition of axon regeneration, presents an important up-regulation, with a fold change of 4.50 and 5.49 in Cx and M respectively. Interestingly, the astrocyte marker *Gfap* (glial fibrillary acidic protein) is up-regulated in Cx and down-regulated in M.

**Figure 10:**
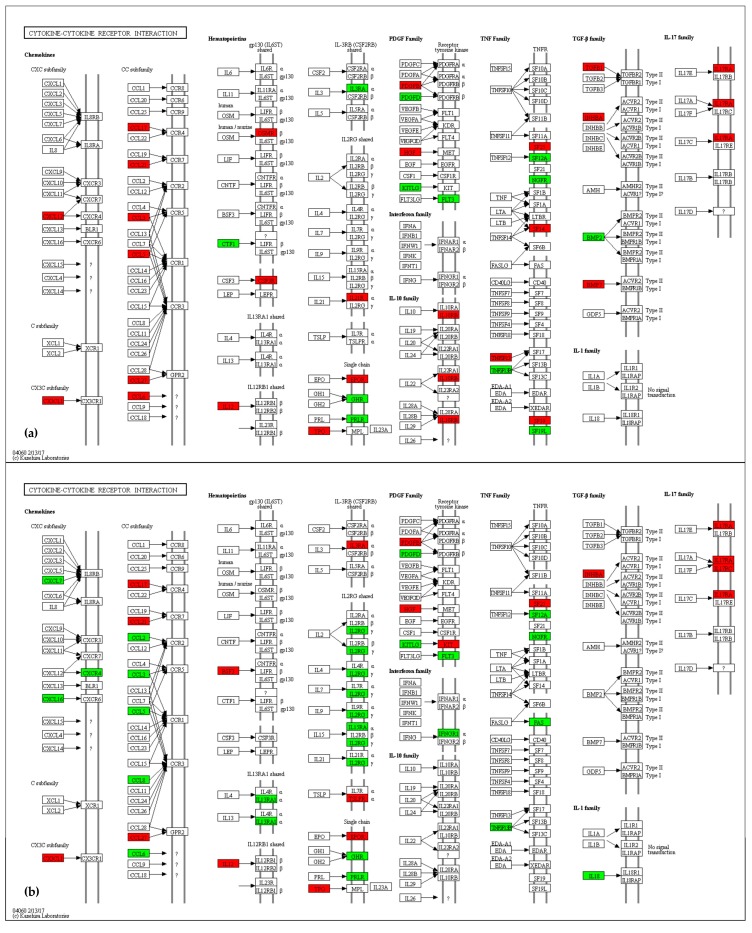
Cytokine-cytokine receptor interaction pathway maps (KEGG—mmu04060): up-regulated (red) and down-regulated (green) genes in the comparison Ids knock-out vs. wild type mice in cerebral cortex (Cx) (**a**) and midbrain/diencephalon/hippocampus (M) (**b**) are shown. Genes are considered differentially expressed for |log2ratio| ≥ 0.7 and *p*-value < 0.05. Black arrow = molecular interaction or activation; ? = unknown gene. For a description of the graphical notation, see Materials and Methods section.

Many genes involved in activation of resident microglia are differentially expressed in the Cx and/or in the M area. Among them, the macrophage phenotypic markers such as *Mpeg1*, the Fc receptors (*Fcer1g*, *Fcgr1*, *Fcgr2b*, *Fcgr3*, *Fcgr4*, and *Fcrls*), *Cd6*8 and *Cd4* antigen, *Mpeg1*, *Lyz1*, *Lyz2*, and *Cyba* are all up-regulated in Cx and down-regulated in M. Accordingly, the factors mostly released from the activated microglia, such as pro-inflammatory cytokines and chemokines, are differentially expressed in Ids-ko brain (Figure 10a,b and in Table S6h). Among them, *Il12a*, *Ccl17*, *Cx3cl1* and *Ccl21b* are up-regulated in both tissues, while *Ccl3*, *Ccl5*, *Ccl6* and *Ccl8* are up-regulated in Cx and down-regulated in M.

Moreover, cathepsins, which are released by activated microglia and associated with the pro-inflammatory response, neuronal death and apoptosis, present gene expression alterations: *Ctsa*, *Ctsd* and *Ctss* are up-regulated in Cx, while *Ctsc*, *Ctsd*, *Ctsh*, *Ctsl*, and *Ctss* are down-regulated in M.

As shown in Table S6h, T cell (*Cd34*, *Cd37*, *Cd4*, *Cd48*, *Cd53*, and *Cd7*) and B cell (*Bcl11a*, *Bcl11b*, *Bcl3*, *Bcl6*, *Bcl9*, and *Bcl9l*) specific genes are also altered, as well as genes involved in the antigen presentation process (*H2* class genes). Finally, in the cerebral cortex an up-regulation of some complement components (*C1qa*, *C1qb*, *C1qc*, *C3ar1*, and *C4b*) is registered, while *C1qa* and *C3ar1* are down-regulated in the M area.

#### 2.4.9. Oxidative Stress

Some genes associated with oxidative damage are altered in Ids-ko cerebral cortex. In particular, we observed an up-regulation of the NADPH-oxidase complex components (*Ncf1*, *Ncf4*, and *Cyba*), nitric oxide synthase 3 (*Nos3*) and glutathione peroxidase 1 (*Gpx1*) genes. The factors involved in antioxidant processes, such as catalase (*Cat*) and superoxide dismutase (*Sod*), are unchanged in both tissues (Table S6i).

#### 2.4.10. Mitochondria

Genes involved in mitochondrial regulation, oxidative phosphorylation and mitochondrial respiratory chain results up-regulated in the Cx and down-regulated in the M areas of the Ids-ko mouse model (Table S6j). We found differentially expressed genes involved in Complex I (*Ndufa12*, *Ndufa6*, *Ndufb5*, *Ndufb6*, *Ndufc1*, *Ndufc2*, *Ndufs3*, *Ndufs4*, and *Ndufv2*), in Complex II (*Sdha* and *Sdhd*), in Complex III (*Uqcrq*, *Uqcrh*, *Uqcr11*, *Uqcrfs1*, and *Uqcrb*), in Complex IV (*Cox5a*, *Cox6a1*, *Cox7a1*, *Cox7a2*, *Cox7b*, *Cox8a*, and *Cox8b*) and in Complex V (*Atp5k* and *Atp6v1f*). In addition, we found an up-regulation in both areas of the genes *Lrrk2*, largely present in the cytoplasm and in the mitochondrial outer membrane and associated with Parkinson’s disease, and *Bbc3*, that cooperates with direct activator proteins to induce mitochondrial outer membrane permeabilization and apoptosis.

#### 2.4.11. Neurodegenerative Disorders

Analysis of the pathways involved in the pathogenesis of the main neurodegenerative disorders (Alzheimer’s, Parkinson’s and Huntington diseases) revealed that several genes are dysregulated in both brain areas of the Ids-ko mouse model (Table S6k). In the Alzheimer’s disease pathway, we found genes up-regulated (*Apbb1*, *Atf6*, *Cacna1d*, *Calm3*, *Casp7*, *Itpr1*, *Lpl*, and *Ppp3ca*) and down-regulated (*Aph1b*, *Bid*, *Cycs*, *Fadd*, *Fas*, and *Nae1*). In addition, the up-regulation of α-synuclein (*Snca*) is noteworthy, as it is involved in the pathogenesis of both Alzheimer’s disease (AD) and Parkinson’s disease (PD). In PD pathway, we found five up-regulated (*Adcy5*, *Adora2a*, *Drd1*, *Lrrk2*, and *Sept5*) and seven down-regulated (*Cycs*, *Gpr37*, *Slc18a2*, *Slc6a3*, *Th*, *Ube2j1*, and *Vdac*) genes. We also found many differentially expressed genes related to the Huntington disease (HD) pathway in both areas of the MPS II mouse model; among these, *Bbc3*, *Bdnf*, *Crebbp*, *Dctn1*, *Dlg4*, *Gpx1*, *Grin1*, *Grin2b*, *Itpr1*, and *Pparg* are up-regulated, while *Dnaic2*, *Dnali1*, *Hap1*, and *Vdac2* are down-regulated. Finally, in this study, we found up-regulated in the Cx and down-regulated in the M area of the Ids-ko mouse most of the genes belonging to the oxidative phosphorylation pathway, which is also involved in AD, PD and HD pathways.

## 3. Discussion

LSDs are very invalidating, often life-threatening diseases due to accumulation of undegraded substrates in most organs, including the brain district in many patients. Understanding the neurological deficit in these diseases has been and remains a very important challenge of the medical-scientific community working in this field.

Cerebral cortex (Cx) is involved in memory, attention, perception, awareness, thought and consciousness, while midbrain, diencephalon and hippocampus (M) are associated with vision, hearing, motor control, sleep/wake, arousal (alertness) and temperature regulation [16].

Converging lines of investigation revealed potentially common pathogenetic mechanisms involved in different neurodegenerative diseases; among them, neuroinflammation and unbalance of calcium homeostasis, in turn connected to oxidative stress, autophagy deficit and defect of the cytoskeleton in vesicular transport. This last may have detrimental implications on the release of neuropeptides in the synaptic cleft, on the transmission of nerve impulses and on axon guidance. These mechanisms are interrelated in a vicious cycle, eventually leading to cell dysfunction and death, the basic molecular mechanisms of which are still unknown [17,18].

Differential expression alterations highlighted by the RNA-seq analysis performed in the present study are mainly concordant in the two brain areas examined for all processes named above, except for neuroinflammation where an altered expression going on opposite directions was observed in the Cx and in the M areas.

### 3.1. Calcium Homeostasis

A dysregulation of the calcium signaling pathway has been previously reported in several LSDs at different levels and with distinct abnormalities [19]. In GM2 gangliosidosis and in Niemann–Pick type A diseases, changes in the sarco-endoplasmic reticulum–Ca^2+^ ATPase (SERCA) activity, with or without sialyl moieties, were reported respectively [20,21]. In addition, enhanced Ca^2+^ release from endoplasmic reticulum has been shown in murine models of Gaucher disease [22].

In the present study, the calcium signaling pathway results severely compromised in both areas of the Ids-ko mouse model, with alteration of transporters of the plasma membrane, mitochondrial and endoplasmic reticulum. In Cx, and even more in M, a dysregulation of *Grin* and *Cacn* gene families, leading to internalization of calcium ions in the cytoplasm, was detected.

In addition, the up-regulation of *Ryr* and *Ip3r* genes leads to an increase in calcium release from the endoplasmic reticulum. Variations of these genes could lead to an increased concentration of cytosolic calcium, in agreement with what observed in other LSDs [19].

### 3.2. Synapse and Neuroactive Ligand—Receptor Interaction

The calcium homeostasis and the circadian rhythm pathways are directly related to cholinergic, dopaminergic and glutamatergic synapse pathways and seem to affect synapse maintenance and strength [23,24]. The signal transmission failure by synapses is the main cause of pathology in adult-onset neurodegenerative diseases and many neurological diseases share similar molecular mechanisms leading to synaptic pathology [25].

Recent studies have demonstrated a synaptic failure also in sphingolipidosis [26], while in NPC1 there are significant defects in synaptic transmission both at glutamatergic and GABAergic synapses, due to an impairment of synaptic vesicle trafficking [27]. Very recently, Sambri and colleagues demonstrated in the MPS IIIA mouse model the direct link between presynaptic maintenance and lysosomal dysfunction and its importance in the LSDs neuropathogenesis [28].

Our study conducted in the MPS II mouse model underlines a heavy dysregulation of all synaptic processes; it involves all types of synapses (cholinergic, glutamatergic, dopaminergic, serotonergic, GABAergic), receptors, neurotransmitters and channels as well as calcium homeostasis. This confirms the close link between synaptic pathology, LSDs and neurodegenerative diseases, and stresses once again the need for a deep study aimed at understanding the molecular mechanisms behind such dysregulation, in order to identify potential new therapeutic targets for the neurological involvement in LSDs.

It is also important to underline that the inflammatory system linked to glial cells can lead to the loss and dysfunction of synapses [29]. Therefore, neuroinflammation in MPS II, as well as in other LSDs, further exacerbates the synaptic pathology and the neurodegeneration in these diseases.

### 3.3. Axon Guidance

An alteration of the axon guidance processes has been so far supposed only for other three LSDs: In the drosophila model for Batten disease [30], in the brain of the MPS VII mouse [8] and in the brain of a mouse model for CLN1 disease [31].

Four main families of signal molecules responsible for axon guidance are known: Netrins, ephrins, semaphorins and slits. These molecules are important also for other processes; for example, *Ntn1* and its receptor *Unc5a* are responsible for the development of several tissues, including the nervous one, both in early stages and in adult life (as repair, neurites directioning and growth of the nervous tissue). In adult tissue, they also seem to be implicated in the migration of stem cells, in the survival of tumor cells and as modulators of inflammation. Mutations or loss of function of *Ntn1* or of netrin receptors result to be lethal in mice [32]. In our work, all four families of molecules and their pathways show gene expression alteration in the MPS II mouse model vs. the wt animal. Given the delicate and important involvement of these molecules in the nervous system development and in the subsequent maintenance of the correct axons directionality, it is not surprising that small changes in expression of these molecules may conduct to alterations of the normal organizational functions and neuronal plasticity, resulting in neurodegeneration. Therefore, the alterations of axon guidance in MPS II may justify the neurological impairment also in the developmental phases of the central and peripheral nervous systems. Furthermore, it is conceivable that the alterations of axon-guidance may compromise the potential replacement of damaged neurons and, at the same time, can hinder the formation of new interconnections between the existing neurons, limiting the regeneration of damaged brain tissue and favoring a process of neurodegeneration, limiting brain plasticity and compromising the learning process.

Moreover, it can be assumed that the alteration of calcium homeostasis may be involved not only in axonal transport but also in axon guidance, as it contributes to membrane depolarization. These processes are crucial also in neural development, as they allow the directioning of axons and the consequent formation of synaptic connections necessary for the proper functioning of the central and peripheral nervous systems.

### 3.4. Circadian Rhythm and Entrainment

The circadian clock in mammals is in the suprachiasmatic nucleus (SCN), a defined group of cells located in the hypothalamus. Alteration in the circadian rhythm was found in different LSDs. In MPS I, Jordan and colleagues demonstrated that the circadian cycle was abnormal in mice relative to heart rate, body temperature and activity [33]. In addition, in MPS III, the circadian production of melatonin seems to be altered, explaining the sleep disorders observed in children [34]. This was confirmed in MPS IIIB mice, where a significant increase in light phase activity was found [35], together with a direct effect of the disorder on the organization and function of the circadian clock in the SCN [36]. Alterations in 17 genes, known to be involved in circadian rhythm, were found also in the brain of MPS VII mouse [8]. Interestingly, Richardson and colleagues have highlighted in Niemann–Pick Type-C and Sandhoff disease that the accumulation of specific metabolites in LSDs may differentially contribute to circadian deregulation at the molecular and behavioral level [37].

Our group has previously shown that the circadian gene expression is altered in fibroblasts from MPS II patients with a direct involvement of the molecular clock machinery in the pathophysiology of cellular derangements [38].

In this study, we confirmed an up-regulation of the circadian rhythm genes in the M area of the MPS II mouse model, but also an alteration of the circadian entrainment and of the circadian Ca^2+^ rhythms.

### 3.5. Regulation of Actin Cytoskeleton

Failure in the control of cytoskeletal signaling can lead to a wrong connection between extracellular stimuli and cellular response. Rho GTPases family plays a key role in actin dynamics and, therefore, also in remodeling of spines and synapses, and their dysfunction lead to cognitive impairments [39].

Proteins linked to Rho GTPase family are also relevant for axon guidance, and aberration of the neuronal outgrowth was observed at the pre-symptomatic stage in the brain of CLN1 mouse model [31]. Cytoskeletal alterations have been observed also in Krabbe, Pompe and Niemann–Pick type C diseases [40,41,42] and in other adult-onset disorders of the nervous system, such as Charcot–Marie–Tooth, Alzheimer’s disease, Parkinson’s disease [26].

The impairment of cytoskeletal regulation found in the MPS II mouse model can therefore be linked to both the strong synaptic impairment and to the axon guidance alteration.

### 3.6. Wnt Signaling

Wnt proteins are implicated in several pathways and their receptor interaction result in a variety of intracellular responses. In the nervous system, Wnt regulates neurogenesis, synaptogenesis and angiogenesis and participates in axon regeneration, astrocyte and glial generation, myelination, blood-brain barrier integrity and inflammation [43].

A deficit in canonical Wnt pathway was also associated to impaired osteoblast differentiation and reduced bone mineralization in the *Gba1* zebrafish model (Gaucher Disease model) [44].

Some neurodegenerative diseases share the same Wnt/β-catenin and peroxisome proliferator-activated receptor (PPAR) γ profile, in which the canonical pathway is down-regulated while PPAR γ is up-regulated (Alzheimer’s disease, bipolar disorder and schizophrenia), other present the opposite gene expression (Parkinson’s disease, Huntington disease amyotrophic lateral sclerosis, multiple sclerosis and Friedreich ataxia) [45].

In this study, we identified an alteration of canonical and non-canonical Wnt signaling in Cx and M areas with a major implication of Wnt/Ca^2+^ pathway. Moreover, in the M area, a greater alteration of the transcriptional factor and of the downstream genes compared to cerebral cortex was detected. These alterations might lead to impairment of axon guidance and neuronal regeneration and plasticity.

### 3.7. Autophagy and CLEAR Network

Implication and importance of autophagy in the pathogenesis of LSDs was shown few years ago [46]. More recently, glycogenoses (Pompe and Danon diseases), multiple sulfatase deficiency (MSD), mucopolysaccharidoses (MPS IIIA, MPS VI), sphingolipidoses (NPC1, NPC2, GM1 gangliosidosis, Gaucher and Fabry disease), mucolipidoses (MLII, MLIII, MLIV) and ceroid lipofuscinoses (CLN10, CLN3) have all shown an impairment in this pathway [47].

A crucial process for the proper induction of autophagy is the coordination of the Atg proteins with other subcellular components, including cytoskeleton, secretory pathway and immunoproteins [48]. In the present study, *Atg* transcripts and other activators of autophagy (*Hif1a*, *Bnip3*, *Eif2a*, *Rragb*, *Camkk2*, and *Tsc2*) are altered in both brain areas examined, suggesting a possible dysregulation of autophagy also in MPS II.

Furthermore, autophagy is important for synaptic and neuronal plasticity as well as for their homeostasis, and dysfunctions of this pathway can be directly related to the onset of neurodegenerative diseases [49]. In fact, most of the protein aggregates associated to a late onset neurodegenerative condition are autophagic substrates [50]. Interestingly, among the data obtained in this RNA-seq study, α-synuclein (*Snca*) transcript, involved in the death of dopaminergic neurons and implicated in several neurodegenerative diseases as Alzheimer disease and Parkinson disease [51], is up-regulated in both Cx and M areas of the Ids-ko mice, supporting an hypothesis of similarity between LSDs and chronic neurodegenerative diseases, as previously proposed by others [52].

### 3.8. Immune and Inflammatory Systems

Lysosomes represent important components in immune cell processes, including autophagy, antigen processing and presentation on MHC, lysosomal degranulation (necessary for T-cell cytotoxicity) and mast cell inflammatory mediator secretion [53]. In fact, an alteration of immune system components has been described in different mouse models of LSDs, such as MPS I, MPS IIIA, MPS IIIB, MPS VII, Gaucher and Niemann–Pick C diseases, which show an increase of macrophage/monocyte functions, activation of resident microglia, astrogliosis, neuronal loss, infiltration of leucocytes, production of inflammatory cytokines [6,54,55,56].

The results obtained in the present study confirm the alteration of the same processes in the Ids-ko mouse brain. Interestingly, these processes seem to be activated only in Cx, remaining unchanged or in some cases impaired, respect to the wt animals, in M.

This different immune response of the two brain areas is also confirmed by the analysis of the 55 genes up-regulated in the Cx and down-regulated in the M area of the ko animal compared to wt. The functional annotation and the pathway analysis of these genes revealed an important enrichment in GO terms and pathways related to the immune system.

A possible explanation could be a difference between Cx and M tissues in immune cell infiltration from the periphery. In fact, this infiltration may be dependent on *Ccl3* (chemokine (C-C motif) ligand 3), as deletion of this chemokine results in a reduction in cellular infiltration, microglial/macrophage pathology, and neuronal apoptosis [53] and *Ccl3* is indeed up-regulated in Cx and down-regulated in M. In addition, *Cxcl12*, a potent chemotactic agent for T cells and monocytes, has the same expression profile.

The alterations in genes specific for T cell, B cell, complement, MHC and immunoglobulins suggest the participation of the adaptive immunity in MPS II pathology, according to the finding in MPS IIIB [57].

### 3.9. Oxidative Stress

Oxidative stress has been considered as possible pathogenetic mechanism in LSDs. However, the central role that it plays in integrating other cellular pathways suggests that in LSDs it is more likely activated as a secondary biochemical pathway, rather being a direct result of accumulation of the primary substrate [58]. In MPS I and MPS IIB mice, an up-regulation of NADPH oxidase complex components was observed, which is probably associated with microglia activation [53]. This is also related with an increased production of superoxide leading to protein, lipid and DNA oxidation. In MPS I, an up-regulation of antioxidative processes, mediated by superoxide dismutase and catalase, also occurs to counteract the oxidative stress and control the toxicity. The present study confirms the involvement of oxidative stress in the Ids-ko mouse cortex. This is consistent with our findings in astrocytes derived from Ids-ko neural stem cells and immunohistological studies in the Ids-ko mouse cortex [59]. The oxidative stress was also documented in MPS II patients [60,61]. The activation of anti-oxidant processes observed in MPS I seems to be absent in Ids-ko brain.

### 3.10. Mitochondria

Mitochondrial impairment is a common feature of LSDs and other neurodegenerative disorders, although the mechanism involved may vary among mitochondrial fragmentation, reduction in mitochondrial respiration and membrane potential, dysregulation of mitochondrial quality control pathways and accumulation of damaged mitochondria [62]. A recent review by Plotegher and Duchen [62] summarizes all the LSDs with some forms of mitochondrial dysfunction and underlines its importance in the pathogenesis of these disease. There can be several causes that lead to mitochondrial dysregulation: the lysosomal impairment that characterizes all LSDs, but also the oxidative stress, autophagy, calcium dyshomeostasis and the accumulation of proteins, such as those characterizing some neurodegenerative diseases (i.e., α-synuclein in Parkinson’s disease) [62].

In a previous study [59], our group found a spotted and disorganized distribution of mitochondria in Ids-ko mouse astrocytes and shorter mitochondrial chain length in MPS II fibroblasts, flanked by the presence of oxidative damage. In this work, we confirmed a mitochondrial dysregulation in the brain of the Ids-ko mouse model, identifying the main impairment at the level of all complexes of the respiratory chain.

### 3.11. Neurodegenerative Disorders

Lysosomal storage disorders and neurodegenerative diseases (Alzheimer’s, Parkinson’s and Huntington diseases) share many features related to the mechanisms that characterize the disease pathogenesis [63]. The first link between LSDs and Parkinson’s disease (PD) have been demonstrated a decade ago for Gaucher disease, and then extended to others, such as Niemann–Pick type I e II (NPC1, NPC2), GM1 and GM2 gangliosidosis, neuronal ceroid lipofuscinoses and Fabry disease [64]. In the last years, common alterations in different pathways have been highlighted, including impairment in the autophagy-lysosome pathway, in calcium homeostasis and oxidative stress, mitochondrial dysfunction and alterations of lipid metabolism [64]. A connection between Alzheimer’s (AD) and NPC1 diseases was also established; they are both characterized by accumulation of amyloid-β (Aβ) peptide and hyper-phosphorylation of tau, involvement of cholesterol and progressive neurodegeneration [65].

In this study, we have detected a possible link of MPS II with AD, PD and Huntington disease (HD). The up-regulation of α-synuclein (*Snca*), identified in both areas of the Ids-ko mouse, is very important as its peptides are a major component of amyloid plaques in the brains of patients with AD, while defects in *Snca* have been involved in the death of dopaminergic neurons in the pathogenesis of PD [63]. In the Ids-ko brains, we found differentially expressed genes involved in different stages of PD pathway, from proteasome dysfunction to mitochondrial impairment and oxidative stress. In the AD pathway, the up-regulation of *Snca* and the resulting accumulation of Aβ may activate caspases, facilitate tau hyper-phosphorylation, disrupting mitochondria function and triggering calcium dysfunction. In the HD pathway, we found differentially expressed genes in the vesicular transport and, above all, in the stages that involve glutamatergic synapses and their receptors (NMDAR), destabilizing Ca^2+^ signaling and leading to mitochondrial dysfunction.

Therefore, it is important to stress that, for MPS II, we also show a link with the late-onset neurodegenerative diseases not only with Alzheimer’s disease and Parkinson’s disease, for which the direct link with other LSDs has already been shown, but also with Huntington disease.

## 4. Materials and Methods

### 4.1. Mice

The C57BL/6 Ids knockout (Ids-ko) mouse, providing the model for MPS II, was a kind gift of J Muenzer (University of North Carolina, Chapel Hill, NC, USA); it was expanded in our animal house and previously characterized [3,4,5,59,66,67,68,69,70,71]. In the experiments here described, Ids-ko and wild-type (wt) mice were housed in light and temperature controlled conditions, with food and water provided ad libitum. All animal care and experimental procedures were conducted according to the national and international animal ethics guidelines. The protocol was approved by the Ethical Committee for Animal Experimentation (CEASA) of the University of Padova (Project Number: 06/2010, Date of approval: 8 February 2010).

### 4.2. Preparation of Brain Tissue Samples

The gene expression profiling by RNA-seq technology was performed in the brains of 7 Ids-ko mice 9 months old and 7 wild-type littermate controls.

Each brain was dissected in two macro-areas: Cx, corresponding to cerebral cortex (neocortex) and M, including the midbrain, the diencephalon (with hypothalamus, thalamus and striatum) and the hippocampus. The dissected areas were placed overnight at 4 °C in RNA later^®^ Tissue Collection (Thermo Fisher Scientific, Waltham, MA, USA) and stored at −80 °C until RNA extraction.

### 4.3. Total RNA Extraction and mRNA Purification

Total RNA was extracted following the standard TRIzol^®^ Reagent protocol (Thermo Fisher Scientific) and quantified using the NanoDrop 1000 spectrophotometer (Thermo Fisher Scientific). Total RNA integrity was assessed by the RNA 6000 Nano Kit and Agilent Bioanalyzer 2100 (Agilent Biotechnologies, Santa Clara, CA, USA). Samples to be analyzed were prepared by pooling 70 µg total RNA for each sample.

mRNA was purified by using the Dynabeads^®^ mRNA Purification Kit (Thermo Fisher Scientific) according to the supplied protocol. The enrichment in mRNA was assessed by using the RNA 6000 Pico Kit and 2100 Bioanalyzer (Agilent Biotechnologies).

### 4.4. SOLiD Sequencing and Sequence Analysis

mRNA was treated and sequenced according to the SOLiD Whole Transcriptome Analysis Kit (Applied Biosystems, Foster City, CA, USA) manufacturers protocols. Briefly, mRNA was fragmented using RNase III and the ligation of the adaptor mix and reverse transcription were performed; libraries were size selected for fragments between 50 and 150 bp, amplified by emulsion PCR and purified using the Ambion flashPAGE Fractionator System, (Thermo Fisher Scientific).

The two areas of the Ids-ko (CxH and MH) and of the wild-type mice (CxWT and MWT) were simultaneously processed in a unique sequencing run by using the SOLiD™ 3 Plus System (Applied Biosystems).

### 4.5. Alignment and Identification of Differentially Expressed Genes

Data retrieved from sequencing have been processed for alignment, identification of differentially expressed genes (DEG) and functional analysis.

The reads obtained were subjected to quality control and aligned on the mouse genome (NCBI37/mm9 assembly) using PASS software (version 2.10; CRIBI Biotechnology Center, University of Padova, Padova, Italy) [72,73] with best-hit, two maximum mismatch and no gap as parameters. For each sample, the number of reads aligned on each gene was determined using a self-written script; only reads aligned to a single gene and only genes with a coverage higher than 50% of their length were taken into consideration.

For the identification of DEG, the following comparisons were considered: CxH vs. CxWT, MH vs. MWT, CxH vs. MH and CxWT vs. MWT; the R package DEGseq (version 1.18, Bioinformatics Division, Tsinghua University, Beijing, China) [74,75] was used with LTR (Likelihood Ration Test) as statistical method.

In each comparison, only genes meeting the following two conditions were considered as differentially expressed: *p*-value < 0.05 and |log2ratio| ≥ 0.7 where ratio is the ratio between the number of unique reads aligned to that gene in the two samples. In the text and in the supplementary figures, the fold change (FC) has also been reported; FC is equal to ratio or to its negative reciprocal for positive and negative values of ratio, respectively.

### 4.6. Gene Ontology and Pathway Analysis

To identify the processes and the biological components most affected by variations of gene expression in the Ids-ko samples compared to controls, the lists of DEG for each area were subjected to a functional enrichment analysis.

Gene Ontology (GO) analysis was performed by using the Database for Annotation, Visualization and Integrated Discovery (DAVID) Bioinformatics Resources 6.8 (Laboratory of Human Retrovirology and Immunoinformatics (LHRI), Frederick, MD, USA) [10,76]. The Functional Annotation Tool with default settings was used; terms were considered significantly enriched for EASE score (modified Fisher exact *p*-value) ≤ 0.001.

Pathway analysis was conducted using the gene-list enrichment tool from KOBAS 3.0 (KEGG Orthology Based Annotation System) (Center for Bioinformatics, Peking University, Beijing, CHN) [11,77] considering as statistically significant pathways those with corrected *p*-value ≤ 0.001.

Kyoto Encyclopedia of Genes and Genomes (KEGG) [78] mapping tool [79] was used to visualize map pathways. The maps shown in Figure 7, Figure 8, Figure 9 and Figure 10 use the KEGG notation, as follows: rectangle = gene product, mostly protein but including RNA; little circle = other molecule, mostly chemical compound; black arrow = molecular interaction or activation; dashed black arrow = indirect effect; white arrow = link to another map; rounded rectangle with text = another map; ? = unknown gene. For a detailed description of KEGG notation, please visit http://www.genome.jp/kegg/document/help_pathway.html.

### 4.7. Data Deposition

All the raw read files were submitted to the GEO database (accession number: GSE95224).

## 5. Conclusions

We here presented a neuro-pathogenetic evaluation of Hunter Syndrome, performed by RNA-seq analysis in two brain areas, cerebral cortex (Cx) and midbrain/diencephalon/hippocampus (M), of the MPS II mouse model.

Cerebral cortex is involved in memory, attention, perception, awareness, thought, and consciousness, while midbrain, diencephalon and hippocampus are associated with vision, hearing, motor control, sleep/wake, arousal (alertness), and temperature regulation [16].

Some of these functions have been described as altered in MPS II patients [80], as well as in other neurological disorders, such as Alzheimer’s, Parkinson’s and Huntington diseases.

Converging lines of investigation have found potentially common pathogenetic mechanisms involved in several neurodegenerative diseases, such as neuroinflammation and unbalance of calcium homeostasis, that are in turn connected to mitochondrial dysfunction, oxidative stress, autophagy deficit and defects of the cytoskeleton in vesicular transport. This last may have detrimental implications on the release of neuropeptides in the synaptic cleft, on the transmission of nerve impulses and on axon guidance [17].

Differentially expressed genes here highlighted mostly showed similar variations in the two brain areas examined for all processes named above, except for neuroinflammation and circadian rhythm. As for neuroinflammation, an altered expression going on opposite directions was observed in the Cx and M areas; we speculate that this could be due to a different grade of leukocyte and monocyte infiltration from the periphery between the two areas. The circadian rhythm is altered only in the M area, likely because the circadian clock in mammals is in the suprachiasmatic nucleus, a defined group of cells located in the hypothalamus, included in the M area.

The RNA-seq analysis was here carried out for the first time on the brain of the MPS II mouse model and allowed us to confirm and expand the involvement of signaling pathways already suspected to be implicated in MPS II physiopathology, such as neuroinflammation, neurodegeneration and circadian rhythm [38,59,69]. This study also allowed to highlight the involvement of pathways so far poorly considered, such as axon guidance, synaptic transmission and Wnt signaling, in the pathogenesis of this disease. Among all these pathways, the Synapses and Neuroactive Ligand–Receptor Interaction pathways present the most up- and down-regulated genes, making us hypothesize that their alteration could play a key role in the neuro-pathophysiology of Hunter Syndrome.

All the involved mechanisms are probably connected to each other in complex relationships that could “feed themselves”, possibly leading to cell dysfunction and death. Deeper targeted analyses of the processes identified, in particular of the synaptic transmission, also investigating the protein changes and the downstream effects, may conduct to a better understanding of MPS II neuropathology.

Moreover, since many LSDs share common pathogenetic cascades, it is not surprising that the processes turned out to be altered in MPS II have already been somehow associated to other LSDs in previous studies, as described above and summarized in Table 2. Interestingly, there are processes that have never been investigated in some diseases, probably because most of the available data are obtained from targeted approaches aimed to evaluate only specific aspects of the pathophysiology. In this context, our results could provide an interesting starting point for further, more targeted studies in other LSDs.

Finally, present evaluations might be useful in the analysis of other, more common neurological diseases sharing the same or part of the pathogenetic pathways, and also to possibly identify common therapeutic strategies.

## Figures and Tables

**Table 1:** Reads sequencing and preprocessing.

Sample ID	Number of Raw Reads	Number of Pcq Reads	Pcq Reads/Raw Reads (%)	Number of Aligned Pcq Reads	Aligned Reads/Pcq Reads (%)
CxH	98,434,960	56,388,301	57.28	38,502,647	68.28
MH	98,716,308	56,155,432	56.89	37,363,194	66.54
CxWT	88,183,367	52,388,424	59.41	33,617,996	64.17
MWT	87,079,859	45,727,298	52.51	28,945,731	63.30

pcq = Passed check quality; CxH = cerebral cortex from Ids knock-out mice; MH = midbrain/diencephalon/hippocampus from Ids knock-out mice; CxWT = cerebral cortex from wild type mice; MWT = midbrain/diencephalon/hippocampus from wild type mice.

**Table 2:** Pathways identified as altered in the present study with the indication of other lysosomal storage disorders (LSDs) in which the same alterations were found. Only pathologies mentioned in this study are reported with relative references.

Pathway	MPS I	MPS III	MPS VII	ML IV	MSD	GM1	GM2	GAUCHER	KRABBE	FABRY	POMPE	NCL/CLN	NPC	OTHER LSDs
Calcium signaling				[19]			[20]	[22]					[19,21]	CHS [19]
Synapse and neuroactive ligand–receptor interaction		[28]											[27]	Sphingolipidosis [25,26]
Axon Guidance			[8]									[31]		Batten [30]
Circadian rhythm and entrainment	[33]	[34,35,36]	[8]				[37]						[37]	
Regulation of actin cytoskeleton									[41]		[42]	[31]	[40]	
Wnt Signaling								[44]						
Autophagy and CLEAR network		[47]		[47]	[47]	[47]		[47]		[47]	[47]		[47]	Danon, ML II, ML III, MPS VI [47]
Immune and inflammatory system	[6,53,55]	[6,53,54,55,57]	[53]				[56]	[56]					[56]	
Oxidative stress	[53]	[53]												
Mitochondria		[62]		[62]	[62]	[62]	[62]	[62]	[62]		[62]	[62]	[62]	MPS IV [62]
Neurodegenerative disorders						[64]	[64]	[64]		[64]		[64]	[64,65]	

Mucopolysaccharidosis = MPS; multiple sulfatase deficiency = MSD; mucolipidosis = ML; GM1 gangliosidosis = GM1; GM2 gangliosidosis = GM2; neuronal ceroid lipofuscinosis = NCL or CLN; Niemann–Pick type C = NPC; Chediak–Higashi syndrome = CHS.

## Supplementary Materials

Supplementary materials can be found at www.mdpi.com/1422-0067/18/5/1072/s1.
